# Including marker x environment interactions improves genomic prediction in red clover (*Trifolium pratense* L.)

**DOI:** 10.3389/fpls.2024.1407609

**Published:** 2024-06-10

**Authors:** Leif Skøt, Michelle M. Nay, Christoph Grieder, Lea A. Frey, Marie Pégard, Linda Öhlund, Helga Amdahl, Jasmina Radovic, Libor Jaluvka, Anna Palmé, Tom Ruttink, David Lloyd, Catherine J. Howarth, Roland Kölliker

**Affiliations:** ^1^ Institute of Biological, Environmental and Rural Sciences, Aberystwyth University, Aberystwyth, United Kingdom; ^2^ Division of Plant Breeding, Fodder Plant Breeding, Agroscope, Zurich, Switzerland; ^3^ Molecular Plant Breeding, Institute of Agricultural Sciences, ETH Zurich, Zurich, Switzerland; ^4^ INRAE P3F, Lusignan, France; ^5^ Lantmännen Lantbruk, Svalöv, Sweden; ^6^ Graminor Breeding Ltd., Bjørke Forsøksgård, Norway; ^7^ Institute for Forage Crops (IKBKS), Kruševac, Serbia; ^8^ DLF Seeds, Hladké Životice, Czechia; ^9^ The Nordic Genetic Resource Centre, Plant Section, Alnarp, Sweden; ^10^ Plant Sciences Unit, Flanders Research Institute for Agriculture, Fisheries and Food, Melle, Belgium; ^11^ Department of Plant Biotechnology and Bioinformatics, Ghent University, Ghent, Belgium; ^12^ Germinal Horizon, Plas Gogerddan, Aberystwyth, United Kingdom

**Keywords:** genomic prediction, marker x environment interaction, population structure, predictive ability, red clover, *trifolium pratense*

## Abstract

Genomic prediction has mostly been used in single environment contexts, largely ignoring genotype x environment interaction, which greatly affects the performance of plants. However, in the last decade, prediction models including marker x environment (MxE) interaction have been developed. We evaluated the potential of genomic prediction in red clover (*Trifolium pratense* L.) using field trial data from five European locations, obtained in the Horizon 2020 EUCLEG project. Three models were compared: (1) single environment (SingleEnv), (2) across environment (AcrossEnv), (3) marker x environment interaction (MxE). Annual dry matter yield (DMY) gave the highest predictive ability (PA). Joint analyses of DMY from years 1 and 2 from each location varied from 0.87 in Britain and Switzerland in year 1, to 0.40 in Serbia in year 2. Overall, crude protein (CP) was predicted poorly. PAs for date of flowering (DOF), however ranged from 0.87 to 0.67 for Britain and Switzerland, respectively. Across the three traits, the MxE model performed best and the AcrossEnv worst, demonstrating that including marker x environment effects can improve genomic prediction in red clover. Leaving out accessions from specific regions or from specific breeders’ material in the cross validation tended to reduce PA, but the magnitude of reduction depended on trait, region and breeders’ material, indicating that population structure contributed to the high PAs observed for DMY and DOF. Testing the genomic estimated breeding values on new phenotypic data from Sweden showed that DMY training data from Britain gave high PAs in both years (0.43–0.76), while DMY training data from Switzerland gave high PAs only for year 1 (0.70–0.87). The genomic predictions we report here underline the potential benefits of incorporating MxE interaction in multi-environment trials and could have perspectives for identifying markers with effects that are stable across environments, and markers with environment-specific effects.

## 1 Introduction

Increased use of high protein legume crops, such as red clover as a forage for ruminants, can aid in reducing the protein deficit in Europe and contribute to sustainable livestock production. Red clover (*Trifolium pratense* L.) is particularly valued in livestock agriculture for its ability to fix atmospheric nitrogen in symbiosis with soil bacteria of the genus *Rhizobium*, reducing the reliance on chemically produced fertiliser. It can enrich the soil and provide companion crops with a supply of fixed nitrogen (Frame et al., 1998). There is also evidence that red clover can improve soil structure and can be a useful component in crop rotations (McKenna et al., 2018). Red clover contains the enzyme polyphenol oxidase (PPO), which catalyses the formation of compounds that can form complexes with and moderate the breakdown of protein in the rumen. This can improve utilisation of nitrogen by the ruminant animals, thereby reducing losses of nitrogen and emissions of methane to the environment (Lee, 2014).

Ready access to chemically produced nitrogen fertiliser, challenges in maintaining the optimal ratio of clover to grass in mixtures, lack of persistency, and insufficient tolerance to grazing still limits the use of red clover in many countries (Lüscher et al., 2014). Additionally, there is an urgent need to produce varieties that are more resilient to climate change.

Genomic selection (GS) is one of the most promising methods of increasing the speed of new plant variety development. The main objectives of this work was to investigate the potential of GS in red clover by comparing different prediction models. The term GS was introduced by Meuwissen et al. (2001) and is based on the following principle: A training population, for which genome-wide molecular marker and phenotypic data are available, is used to estimate the effect of each marker on each phenotype. This information is then used in a test population with only the molecular marker information available to determine a genomic estimated breeding value (GEBV), which in turn is used to select individuals for further crossing in a breeding programme. GS or genomic prediction (GP) has shown its utility particularly in dairy cattle breeding (Schefers and Weigel, 2012; Hayes et al., 2013b; Wiggans et al., 2017). It is also being incorporated in plant breeding, notably in the major cereals such as maize (*Zea mays* L.) (Zhao et al., 2012), rice (*Oryza sativa* L.) (Cui et al., 2020; Xu et al., 2021), wheat (*Triticum* spp.) (Bassi et al., 2016; Juliana et al., 2020), barley (*Hordeum vulgare* L.) (Puglisi et al., 2021) and oats (*Avena sativa* L.) (Campbell et al., 2021).

Among forage crops, GP has been most intensively studied in perennial ryegrass (*Lolium perenne* L.) (Hayes et al., 2013a; Forster et al., 2014; Fè et al., 2015, 2016; Grinberg et al., 2016; Lin et al., 2016; Byrne et al., 2017; Lin et al., 2017; Arojju et al., 2018; Cericola et al., 2018; Faville et al., 2018; Pembleton et al., 2018; Arojju et al., 2020; Faville et al., 2020; Barre et al., 2022). Many of those studies indicate that predictive abilities (PAs) are highest for traits with high heritability such as heading date, and disease resistance, while PAs for DMY were moderate to low.

Genomic prediction in forage legumes has received less attention than in ryegrass. However, some work has been carried out on DMY in alfalfa (*Medicago sativa* L.) (Annicchiarico et al., 2015; Li et al., 2015; Annicchiarico et al., 2017), and white clover (*T. repens* L.) (Griffiths et al., 2022) with encouraging results. However, the perennialism and often outbreeding nature of most forage crops present some challenges. Varieties are usually synthetic populations derived from crossing a small number of parents after they have been selected among and/or within families based on observation of performance of their half-sib progeny (Barre et al., 2022). Either way, the varieties are populations consisting of related, but genetically distinct and heterozygous individuals. Genomic prediction models are thus often based on genotypes of the parents, and phenotypes of their half-sib progeny (Annicchiarico et al., 2015; Grinberg et al., 2016; Arojju et al., 2020), or based on genotypes and phenotypes of spaced plants for suitable traits (Byrne et al., 2017; Arojju et al., 2018).

An alternative is to genotype pools of plants at the level of family or population using methods such as Genotyping-By-Sequencing (GBS) (Elshire et al., 2011) or RAD-seq (Baird et al., 2008). Allele frequency data on a population or family level allows predictions to be made based on genotypic and phenotypic data from the same population, not based on genotypic data from parents and phenotypic data from their progenies. It has been used in ryegrass for prediction of flowering time, crown rust resistance, seed yield and fructan content (Byrne et al., 2013; Fè et al., 2016; Cericola et al., 2018). Recently, GP was also successfully explored in alfalfa using pooled samples for genotyping (Pégard et al., 2023). PA was above 0.75 for plant height and dormancy in some years, demonstrating the potential of GP for some traits.

Pooled genotyping has been used in red clover to identify regions under selection for survival in the field (Ergon et al., 2019), and for genome-wide identification of loci involved in timing of stem elongation and freezing tolerance (Ergon et al., 2022; Zanotto et al., 2023). Allele frequency data from pooled samples compared well with those obtained by genotyping individuals from the same population (Ergon et al., 2019).

GP has mostly been used in single environment contexts, largely ignoring genotype by environment interaction (GxE), i.e. effects of location and year, that greatly affect crop performance. However, in the last decade, models incorporating GxE have been described (Burgueño et al., 2012; Heslot et al., 2014; Jarquín et al., 2014; Lopez-Cruz et al., 2015). While Juliana et al. (2020) reported no advantage of using GxE models, many other studies found that it increased PA, e.g. in wheat (Lopez-Cruz et al., 2015; Crossa et al., 2016; Sukumaran et al., 2018), maize (Bandeira E Sousa et al., 2017), barley (Puglisi et al., 2021) and sesame (*Sesamum indicum* L.) (Sabag et al., 2023).

This work was aimed at investigating whether incorporating marker by environment interaction effects (MxE) in the prediction models could be advantageous, compared to analysis trait by trait. The focus was not on GP in a breeding programme. More specifically the objective was to assess GP for DMY, CP content and DOF in a diverse panel of red clover accessions using different prediction models. GxE was incorporated using the strategy described by Lopez-Cruz et al. (2015), as this often performed best in the investigations described above. Three models were compared: (1) SingleEnv, where PA was measured separately for each environment, (2) AcrossEnv, where marker effects were an average across environments, and (3) MxE where marker effects were divided into those that were stable across environments, and those that were environment-specific. Finally, we attempted to validate the models by predicting phenotypes derived from independent experiments.

## 2 Materials and methods

### 2.1 Plant materials

The data on which much of this work is based were obtained through the EUCLEG project (Horizon 2020 Programme for Research & Innovation, grant agreement no. 727312; http://www.eucleg.eu) and have been described in detail previously (Nay et al., 2023). Briefly, field experiments with 400 accessions were established in Switzerland (CHE: 47.480°N, 8.904°E) and in Czechia (CZE: 49.690°N, 17.960°E) in a partially replicated (p-rep) design. The field experiment was abandoned after year 1 in CZE, so no data from year 2 were available from this location. Field experiments with approximately 100 accessions were established in Britain (GBR: 52.427°N, 4.020°W) with 100, Norway (NOR: 60.757°N, 11.203°E) with 109, and Serbia (SRB: 43.583°N, 21.206°E) with 100. For the latter three locations, twenty accessions were included at every site, and between 12 and 17 of the remaining accessions overlapped between pairs of locations (**Supplementary Table S1**). From all five locations annual DMY from year 1 and 2 (DMY1 and DMY2), CP content from cut 1 and cut 2 in year 1 (CP1 and CP2), and date of flowering (DOF) from year 1 were used here. **Supplementary Table S1** contains more detailed information about the accessions used. A p-rep design was also used in GBR. For the p-rep trials there were no complete blocks, so that each row and column was an incomplete block (IB1 and IB2, respectively). The trials from NOR and SRB were Alpha designs with two complete blocks containing all the accessions. Observation rows were used to record DOF with two complete blocks at all sites.

To test the GEBVs derived from the field trials described above, we used data obtained from field trials at three additional sites, all in Sweden. A total of 49 accessions were included in these field trials in which a randomised complete block design (RCBD) was used with two replicates (blocks) at Bjertorp (BJT: 58.250°N, 13.117°E) and Kölbäck (KLB: 58.433°N, 15.250°E), but three replicates at Svalöv (SVA: 55.900°N, 13.100°E) (**Supplementary Figure S1**). DMY1, DMY2, CP1 and CP2 data were obtained. Of the 49 accessions, 42 were part of the EUCLEG panel, so only those were genotyped.

### 2.2 Genotypic data

GBS was used to obtain allele frequency data from a total of 400 red clover accessions as previously described (Frey et al., 2022; Nay et al., 2023). A total of 200 seedlings per accession were germinated in a greenhouse. The leaf at the one-leaf stage was harvested from each seedling, and the leaves from each accession were combined. DNA was extracted from each sample using the QIAGEN DNeasy 96 Plant kit (QIAGEN, Citylabs 2.0, Manchester M13 0BH, UK), and the concentration normalized to 20 ng µl^-1^. Genotyping was carried out by LGC Genomics (Berlin, Germany) using a *Pst*I-*Mse*I double-digest pool-GBS method followed by PE-150 Illumina sequencing. Sequencing data covered 10,609 unique loci with an average read depth of 288. SNP calling and calculations of allele frequencies were done as described in Keep et al. (2020). The parameters used are described in detail in the **supplementary methods** of Frey et al. (2022). SNPs were retained if allele frequencies of at least 10 accessions were between 0.05 and 0.95, and if mean allele frequencies across all accessions were between 0.05 and 0.95. SNP positions with more than 5% missing values were discarded. The remaining missing values were imputed by the mean allele frequency across all accessions. After quality control and filtering, allele frequency data of a total of 20,156 SNP markers in 392 accessions were retained.

### 2.3 Experimental design and phenotypic data

The phenotypic data from all locations except Sweden were analysed using the methods described earlier (Nay et al., 2023). The data used in the present work were based on scaled and normalised values of the best linear unbiased estimates (BLUEs) from each location separately. The data were analysed as seen in Equation 1 using the following model:


(1) $$\begin{array}{c} y_{imno}=\;\mu +\;{ɡ}_{i}+\;b_{m}+\;ib1_{n}+ib2_{o}+\;\epsilon_{imno} \end{array}$$

where *y_imno_* is the phenotype on a single plot, *μ* is the overall mean, *g_i_* is the effect of the i^th^ accession, *b_m_* is the effect of the *m^th^* block, *ib*1_n_ is the effect of incomplete block 1 (i.e. row n), *ib*2*_o_* is the effect of incomplete block 2 (i.e. column o) and *ϵ_imno_* is the residual error. For the p-rep designs (CZE, CHE and GBR) the block term was omitted, while for NOR the *ib*2*_o_* term was omitted. Data were available only from one complete block in SRB, so no separate analysis was performed with data from this location. A linear mixed model analysis was performed with *ib*1*_n_* and *ib*2*_o_* as random effects, while *g_i_* (and *b_m_* where relevant) were treated as fixed effects to obtain the BLUE values for each accession. Best linear unbiased predictions (BLUP) values were subsequently obtained by treating *g_i_* as a random effect. These were used to estimate heritabilities using the method of Walsh and Lynch (2018) by regressing BLUP on BLUE for each trait. The relevant correlations and heritability values are reproduced in **Supplementary Table S2**.

The layout of the field trials in Sweden was a randomised complete block design (RCBD), where BJT and KLB had two replicates and SVA three replicates or blocks. BLUE values of accessions from each location in Sweden were obtained using the following model:


(2) $$\begin{array}{c} y_{ij}=\;\mu +\;{ɡ}_{i}+b_{j}\;+\varepsilon_{ij} \end{array}$$

where *y_ij_* is the phenotypic value of the i^th^ accession in the j^th^ block, *μ* is the overall mean, *g_i_* is the fixed effect of the *i^th^* accession, *b_j_* is the random effect of the *j^th^* block, and *ε_ij_* is the residual (Equation 2). The data were also analysed by having the accessions as a random effect to obtain best linear unbiased predictions (BLUP).The broad sense heritability was calculated as follows:


(3) $$\begin{array}{c} h_{b}^{2}=\;\frac{\sigma_{ɡ}^{2}}{\sigma_{ɡ}^{2}+\frac{\sigma_{\varepsilon }^{2}}{n_{r}}}, \end{array}$$

where $\sigma_{g}^{2}$ is the genetic variance, $\sigma_{\varepsilon }^{2}$ is the residual variance and *n_r_* is the number of replicates (Equation 3). The raw phenotypic data and the heritability values are given in **Supplementary Tables S3** and **S4**.

### 2.4 Genomic predictions

Three models for GPs were used, the first is a single environment (SingleEnv) strategy, in which separate analyses were carried out for each environment. A linear model was used as follows:


(4) $$\begin{array}{c} y_{j}=\;1\mu_{j}+X_{j}\;\beta_{j}+\;\varepsilon_{j} \end{array}$$

where ***y_j_*** is a vector of phenotypes from the j^th^ environment, ***μ_j_*** is the overall mean, ***X_j_*** is a matrix of marker allele frequencies, all centred (by subtracting the mean allele frequency) and standardised (by dividing by the standard deviation), and ***β_j_*** is a vector of marker effects, N(**0**, $I\sigma_{\beta j}^{2}$ ). Finally, ***ε_j_*** is the residual, N(**0**, $I\sigma_{\varepsilon j}^{2}$ ). The model (Equation 4) assumes equal variance across environments. For genomic best linear unbiased prediction (GBLUP) this can be rewritten so that ***u_j_* = *X_j_ β_j_***, so that ***u_j_*** ~ N(**0**, $G_{j}\sigma_{uj}^{2}$ ), with $G_{j}=\frac{X_{j}X_{j}^{'}}{p}$ , where *p* is the number of markers, and $\sigma_{uj}^{2}$ is the marker variance for the *j^th^* environment.

The second strategy assumes that the marker effects are the same across the environments being compared (AcrossEnv). This means that the linear model, where *n* environments are compared, becomes:


(5) $$\begin{array}{c} \left[\begin{array}{c} y_{1}\\ y_{2}\\ \vdots \\ y_{n} \end{array}\right]=\;\left[\begin{array}{c} 1{µ}_{1}\\ 1{µ}_{2}\\ \vdots \\ 1{µ}_{n} \end{array}\right]+\;\left[\begin{array}{c} u_{1}\\ u_{2}\\ \vdots \\ u_{n} \end{array}\right]+\;\left[\begin{array}{c} \varepsilon_{1}\\ \varepsilon_{2}\\ \vdots \\ \varepsilon_{n} \end{array}\right] \end{array}$$

with ***u*** ***_j_** =* ***X_j_β*** and $u=(u_{1}^{'},u_{2}^{'},\text{...}u_{u}^{'})'$ ~ *N*(**0**, ***G_0_***, $\sigma_{u}^{2}$ ), for which $\sigma_{u}^{2}$ is the variance for the main genetic effects across environments (Equation 5). ***G_0_*** is a marker-derived genomic relationship matrix (Equation 6).


(6) $$G_{0}=\;\left[\begin{array}{cccc} X_{1}X_{1}^{'} & X_{1}X_{2}^{'} & \cdots & X_{1}X_{n}^{'}\\ X_{2}X_{1}^{'} & X_{2}X_{2}^{'} & \ldots & X_{2}X_{n}^{'}\\ \vdots & \vdots & \ddots & \vdots \\ X_{n}X_{1}^{'} & X_{n}X_{2}^{'} & \ldots & X_{n}X_{n}^{'} \end{array}\right]/p$$

In the work described here, the marker matrices ***X***_1_ ***, X***_2_ ***…X_n_*** are identical for each multi-environment analysis, so ***X*** *_j_* reduces to ***X***.

The third strategy incorporates marker x environment (MxE) interaction effects. This is done by splitting the effect of the *k^th^* marker on the *j^th^* environment *β_jk_* into two parts, one which is the same in all environments *b_0k_* (***b_0_* ~** *N*(**0**, ***I*** $\sigma_{b_{0}}^{2}$ )) and one which is specific for each environment *b_jk_* (***b_j_*** ~ *N*(**0**, ***I*** $\sigma_{b_{j}}^{2}$ )). For GBLUP the model then can be written as shown in Equation 7:


(7) $$\begin{array}{c} y=\;µ+\;u_{0}+\;u_{1}+\;\varepsilon_{1} \end{array}$$

where ***y*** = (***y***_1_ + ***y***_2_ + … ***y*** *_n_*)’ is the response vector for which ***y*** *_j_* is the vector of observations of the accessions in the *j^th^* environment, and.


$$\mu =\;\left[\begin{array}{c} 1{µ}_{1}\\ 1{µ}_{2}\\ \vdots \\ 1{µ}_{n} \end{array}\right],\;u_{0}=\;\left[\begin{array}{c} X_{1}\\ X_{2}\\ \vdots \\ X_{n} \end{array}\right]b_{0},\;u_{1}=\;\left[\begin{array}{cccc} X_{1} & 0 & \cdots & 0\\ 0 & X_{2} & \ldots & 0\\ \vdots & \vdots & \ddots & \vdots \\ 0 & 0 & \ldots & X_{n} \end{array}\right]\left[\begin{array}{c} b_{1}\\ b_{2}\\ \vdots \\ b_{n} \end{array}\right]$$

in which µ is the overall mean, ***u***_0_ ~ *N*(**0**, ***G_0_*** $\sigma_{uo}^{2}$ ), for which $\sigma_{uo}^{2}$ represents the variance of the common effects across environments as described above for the AcrossEnv model, ***u***_1_ ~ *N*(**0**, ***G_1_***), $\varepsilon ∼N(0,\;I\sigma_{\varepsilon }^{2})$ . ***G_1_*** is a relationship matrix, which, as seen in Equation 8, captures the environment specific effects:


(8) $$G_{1}=\left[\begin{array}{cccc} \sigma_{u1}^{2}X_{1}X_{1}^{'}\; & \;0 & \ldots & 0\\ 0 & \sigma_{u2}^{2}X_{2}X_{2}^{'}\; & \ldots & 0\\ \vdots & \vdots & \ddots & \vdots \\ 0 & 0 & \ldots & \sigma_{un}^{2}X_{n}X_{n}^{'}\; \end{array}\right]/p$$

The Bayesian generalised linear regression (BGLR) package (Pérez and De Los Campos, 2014) and the code described by Lopez-Cruz et al. (2015) were used for calculations. The number of iterations was set to 25,000 with 2,500 for burn-in.

Two cross validation methods, CV1 and CV2 as described by Lopez-Cruz et al. (2015), were applied. Briefly, the PAs were based on the mean of 50 random partitions between the training set and test set with a default split of 70% to 30%, respectively. CV1: test accessions have not been assessed in any of the environments. Thus for multi-environmental analyses the test set consisted of the same accessions from all environments in the analysis. CV2: test accessions have been evaluated in some, but not all environments, and the test sets do not overlap. The methods are illustrated for a two-environment scenario in **Supplementary Figure S2**.

The SingleEnv analysis is straightforward as it produces PA values for each environment separately. For the AcrossEnv and MxE methods, multi-location analysis of trial sites was not possible for GBR, NOR and SRB, as they shared only few accessions. Only comparisons between CHE and CZE were meaningful as they shared all 392 accessions. Since the field experiment in CZE was abandoned after the first year, only data from year 1 could be included from this location. Most of the multi-environment analyses were thus pair-wise comparisons between DMY data from year 1 and 2 at the same location, or between CP data from cut1 and cut 2 in year 1. The multi-location analyses included a pair-wise comparison of DOF in CHE and CZE, a three-way comparison of CHE_DMY1, CHE_DMY2 and CZE_DMY1, a pairwise analysis of CHE_DMY1 and CZE_DMY1, and finally a four-way analysis of the CP content at cut1 and 2 at both locations. The AcrossEnv and the MxE strategies produces PA values for each environment, based on the joint analysis of the environments. It should also be noted that for the SingleEnv analyses the two CV methods are equivalent, as they both use 50 randomly selected training/test set combinations.

A third CV method was used which either left one of the five regions out from the training set, and used it as test set, or left one of the six breeders’ material out and used that as test set (**Supplementary Table S1**). The number of accessions per region varied between 16 and 142, and per breeders’ material it ranged from 18 to 61. For comparison the CV1 method was used to remove 142 accessions randomly (comparing with regions), or 61 accessions (comparison with breeders’ material) from the training set. For DMY the MxE model was used for the data from CHE year 1 and 2 to obtain the GEBVs. For DOF the MxE model was used in the joint analysis of data from CHE and CZE, year 1.

The PA data from the output were analysed using analysis of variance (ANOVA) to identify significant differences between environments, models and cross validation methods. One-way ANOVA was used to assess the effect of each factor, and three-way ANOVA to assess the combined effect of all three factors. The R statistical software was used for this (R Core Team, 2023).

## 3 Results

Initially, a variance component analysis was performed for each phenotype to get information on how the SingleEnv, the AcrossEnv and MxE models fitted the data. Secondly, GP analyses were performed to compare the performance of the three models. The multi-environment analyses included joint analyses of pairs of environments at the same location (year 1 and 2) for DMY, and joint analyses of the two locations CHE (year 1 and 2) and CZE in year 1. For CP content joint analyses of pairs of environments at the same location (cut 1 and cut 2 in year 1) were performed, and joint analyses of the two locations CHE and CZE at cut 1 and cut 2.

### 3.1 Variance component analysis for DMY

The variance component estimations for the annual DMY data derived from SingleEnv analyses varied considerably among the environments (**Table 1**). The residual variance was much higher for CZE than CHE, hence the proportion of the variance explained by the markers was considerably higher for CHE. In the multi-environment analyses, the *R^2 ^* values were generally higher for the MxE model compared to the AcrossEnv model. In the joint analysis of CZE.1, CHE.1 and CHE. 2, the MxE interaction variance was very high for CZE.1. Consequently, the *R^2 ^* values were very high for the MxE model. The specific-environment variance for CZE.1 contributed 84.3% of the total variance (**Supplementary Figure S3**). For the other environments the proportions were between 15.2% and 41.9%. In all cases the *R^2 ^* values were higher for the MxE model than for the AcrossEnv model (**Table 1**). The DMY data from GBR, NOR and SRB were based on approximately 100 accessions. Due to the scant overlap between accessions at those three locations, multi-environment analyses were thus carried out only between the two years at each site, not between the locations. At all three of those locations the genetic effects in the MxE model accounted for a higher proportion of total variance than the AcrossEnv model (**Table 1**).

**Table 1:** Variance component estimation (SD) of annual DMY from field trials in Czechia (CZE), Switzerland (CHE), Britain (GBR), Norway (NOR) and Serbia (SRB) using the SingleEnv, AcrossEnv and the MxE interaction models.

	SingleEnv model			AcrossEnv model			MxE model			
Environment	Residual variance (*σ^2^_E_*)	Marker variance (*σ^2^_U_*)	*R^2^*	Residual variance (*σ^2^_E_*)	Marker variance (*σ^2^_U0_*)	*R^2^*	Residual variance (*σ^2^_E_*)	Marker variance (*σ^2^_U0_*)	M x E interaction (*σ^2^_U1_*)	*R^2^*
CZE_DMY1	0.737(0.068)	0.301(0.072)	0.289(0.057)	*n.a.*	*n.a.*	*n.a.*	*n.a.*	*n.a.*	*n.a.*	*n.a.*
CHE_DMY1	0.111(0.014)	0.288(0.034)	0.722(0.040)	*n.a.*	*n.a.*	*n.a.*	*n.a.*	*n.a.*	*n.a.*	*n.a.*
CHE_DMY2	0.170(0.026)	0.512(0.069)	0.748(0047)	*n.a.*	*n.a.*	*n.a.*	*n.a.*	*n.a.*	*n.a.*	*n.a.*
GBR_DMY1	0.172(0.040)	0.490(0.093)	0.737(0.060)	*n.a.*	*n.a.*	*n.a.*	*n.a.*	*n.a.*	*n.a.*	*n.a.*
GBR_DMY2	0.187(0.044)	0.492(0.098)	0.721(0.065)	*n.a.*	*n.a.*	*n.a.*	*n.a.*	*n.a.*	*n.a.*	*n.a.*
NOR_DMY1	0.386(0.102)	0.592(0.161)	0.598(0.108)	*n.a.*	*n.a.*	*n.a.*	*n.a.*	*n.a.*	*n.a.*	*n.a.*
NOR_DMY2	0.375(0.133)	0.604(0.207)	0.610(0.130)	*n.a.*	*n.a.*	*n.a.*	*n.a.*	*n.a.*	*n.a.*	*n.a.*
SRB_DMY1	0.526(0.115)	0.491(0.149)	0.481(0.105)	*n.a.*	*n.a.*	*n.a.*	*n.a.*	*n.a.*	*n.a.*	*n.a.*
SRB_DMY2	0.430(0.114)	0.607(0.179)	0.578(0.111)	*n.a.*	*n.a.*	*n.a.*	*n.a.*	*n.a.*	*n.a.*	*n.a.*
CZE_DMY1 & CHE_DMY1	*n.a*	*n.a*	*n.a*	0.533(0.033)	0.212(0.032)	0.283(0.035)	0.178(0.028)	0.114(0.041)	1.611(0.267) 0.137(0.042)	0.903(0.032) 0.585(0.053)
CHE_DMY1 & CHE_DMY2	*n.a*	*n.a*	*n.a*	0.259(0.016)	0.271(0.034)	0.510(0.036)	0.101(0.011)	0.262(0.034)	0.066(0.017) 0.290(0.048)	0.763(0.030) 0.844(0.023)
CZE_DMY1 & CHE DMY1 & CHE DMY2	*n.a*	*n.a*	*n.a*	0.511(0.023)	0.198(0.027)	0.278(0.030)	0.138(0.016)	0.218(0.032	1.910(0.236) 0.065(0.018) 0.257(0.050)	0.938(0.016) 0.671(0.039) 0.773(0.036)
GBR_DMY1 & GBR_DMY2	*n.a*	*n.a*	*n.a*	0.149(0.020)	0.444(0.077)	0.745(0.043)	0.100(0.017)	0.411(0.078)	0.094(0.030) 0.095(0.032)	0.833(0.035) 0.833(0.035)
NOR_DMY1 & NOR_DMY2	*n.a*	*n.a*	*n.a*	0.544(0.087)	0.399(0.122)	0.417(0.095)	0.357(0.086)	0.317(0.152)	0.245(0.122) 0.269(0.130)	0.605(0.104) 0.609(0.111)
SRB_DMY1 & SRB_DMY2	*n.a*	*n.a*	*n.a*	0.512(0.072)	0.465(0.148)	0.470(0.086)	0.361(0.069)	0.486(0.148)	0.152(0.070) 0.179(0.080)	0.633(0.087) 0.642(0.084)

*R^2^* of models was calculated as the ratio of the sum of the main and interaction variance relative to the sum of the residual, the main and the interaction variance. n.a., not applicable.

### 3.2 Variance component analysis for CP content

The residual variance proportions were generally high for CP content, which resulted in lower *R^2 ^* values than for DMY in all three models (**Table 2**). Again, the variance explained by the MxE model was higher than for the AcrossEnv model. These results are generally consistent with the low heritability recorded for CP content (**Supplementary Table S2**; Nay et al., 2023). The exception was NOR, where the *R^2 ^* values and heritability were higher than for the other locations, at least in the joint analysis (**Table 2**). **Supplementary Figure S4** underlines this, showing that except for NOR, the residual variance contributes over 70% of the total variance, and the main effect variance was 10.1% or lower. The MxE model thus explains little of the phenotypic variation for this trait.

**Table 2:** Variance component estimation (SD) of CP content of cut 1 (CP1) and cut 2 (CP2) in year 1 from field trials in Czechia (CZE), Switzerland (CHE), Britain (GBR), Norway (NOR) and Serbia (SRB) using the SingleEnv, AcrossEnv and the MxE interaction models.

	SingleEnv			AcrossEnv model			MxE model			
Environment	Residual variance (*σ^2^_E_*)	Marker variance (*σ^2^_U_*)	*R^2^*	Residual variance (*σ^2^_E_*)	Marker variance (*σ^2^_U0_*)	*R^2^*	Residual variance (*σ^2^_E_*)	Marker variance (*σ^2^_U0_*)	M x E interaction (*σ^2^_U1_*)	*R^2^*
CZE_CP1	0.863(0.076)	0.247(0.067)	0.221(0.053)	*n.a.*	*n.a.*	*n.a.*	*n.a.*	*n.a.*	*n.a.*	*n.a.*
CZE_CP2	0.854(0.071)	0.206(0.053)	0.194(0.044)	*n.a.*	*n.a.*	*n.a.*	*n.a.*	*n.a.*	*n.a.*	*n.a*
CHE_CP1	0.703(0.072)	0.349(0.087)	0.330(0.067)	*n.a.*	*n.a.*	*n.a.*	*n.a.*	*n.a.*	*n.a.*	*n.a.*
CHE_CP2	0.828(0.071)	0.249(0.063)	0.230(0.050)	*n.a.*	*n.a.*	*n.a.*	*n.a.*	*n.a.*	*n.a.*	*n.a.*
GBR_CP1	0.522(0.127)	0.579(0.186)	0.737(0.060)	*n.a.*	*n.a.*	*n.a.*	*n.a.*	*n.a.*	*n.a.*	*n.a.*
GBR_CP2	0.786(0.148)	0.397(0.147)	0.333(0.099)	*n.a.*	*n.a.*	*n.a.*	*n.a.*	*n.a.*	*n.a.*	*n.a.*
NOR_CP1	0.596(0.129)	0.472(0.158)	0.439(0.114)	*n.a.*	*n.a.*	*n.a.*	*n.a.*	*n.a.*	*n.a.*	*n.a.*
NOR_CP2	0.226(0.049)	0.425(0.087)	0.650(0.074)	*n.a.*	*n.a.*	*n.a.*	*n.a.*	*n.a.*	*n.a.*	*n.a.*
SRB_CP1	0.692(0.149)	0.465(0.170)	0.398(0.115)	*n.a.*	*n.a.*	*n.a.*	*n.a.*	*n.a.*	*n.a.*	*n.a.*
SRB_CP2	0.818(0.151)	0.379(0.139)	0.315(0.095)	*n.a.*	*n.a.*	*n.a.*	*n.a.*	*n.a.*	*n.a.*	*n.a.*
CZE_CP1 & CZE_CP2	*n.a*	*n.a*	*n.a*	0.774(0.059)	0.295(0.098)	0.274(0.075)	0.766(0.065)	0.243(0.105)	0.094(0.035) 0.082(0.028)	0.301(0.079) 0.294(0.079)
CHE_CP1 & CHE_CP2	*n.a*	*n.a*	*n.a*	0.864(0.050)	0.136(0.038)	0.135(0.034)	0.796(0.050)	0.088(0.036)	0.151(0.057) 0.115(0.042)	0.230(0.051) 0.203(0.045)
CZE_CP1 & CHE CP1 & CZE CP2 & CHE_CP2	*n.a*	*n.a*	*n.a*	0.935(0.037)	0.065(0.021)	0.065(0.020)	0.859(0.037)	0.040(0.021)	0.098(0.043) 0.145(0.054) 0.077(0.030) 0.113(0.042)	0.137(0.042) 0.176(0.045) 0.119(0.033) 0.150(0.042)
GBR_CP1 GBR_CP2	*n.a*	*n.a*	*n.a*	0.932(0.101)	0.115(0.044)	0.129(0.047)	0.769(0.100)	0.072(0.035)	0.234(0.112) 0.182(0.096)	0.281(0.084) 0.245(0.079)
NOR_CP1 NOR_CP2	*n.a*	*n.a*	*n.a*	0.577(0.065)	0.250(0.066)	0.302(0.063)	0.326(0.072)	0.174(0.071)	0.479(0.208) 0.190(0.072)	0.651(0.113) 0.527(0.083)
SRB_CP1 SRB_CP2	*n.a*	*n.a*	*n.a*	0.882(0.102)	0.162(0.075)	0.154(0.064)	0.809(0.108)	0.108(0.065)	0.176(0.093) 0.156(0.079)	0.259(0.084) 0.245(0.082)

*R^2^* of models was calculated as the ratio of the sum of the main and the interaction variance relative to the sum of the residual, the main and the interaction variance. n.a., not applicable.

### 3.3 Variance component analysis for DOF

DOF was recorded during year 1, which meant that only one joint analysis was possible, namely between the locations CHE and CZE. The variance explained by the genetic markers was higher than for the CP content, but lower than for DMY (**Table 3**). In the SingleEnv analysis, the proportion of the variance explained by markers was higher for CZE than for CHE. For the MxE model the proportions of main effect, specific environmental effect and residual variance were similar for the two locations (**Supplementary Figure S3**). In the SingleEnv analysis for GBR, NOR and SRB the proportion of the variance explained by the genetic markers was similar for the three locations, and somewhat higher than the corresponding ones from the SingleEnv analysis of the CZE and CHE data (**Table 3**).

**Table 3:** Variance component estimation (SD) of flowering time (DOF) from field trials in Czechia (CZE), Switzerland (CHE), Britain (GBR), Norway (NOR) and Serbia (SRB) using the SingleEnv, AcrossEnv and the MxE interaction models.

	SingleEnv model			AcrossEnv model			MxE model			
Environment	Residual variance	Marker variance	*R^2^*	Residual variance (*σ^2^_E_*)	Marker variance (*σ^2^_U0_*)	*R^2^*	Residual variance (*σ^2^_E_*)	Marker variance (*σ^2^_U0_*)	M x E interaction (*σ^2^_U1_*)	*R^2^*
CZE_DOF	0.346(0.041)	0.385(0.069)	0.525(0.063)	*n.a.*	*n.a.*	*n.a.*	*n.a.*	*n.a.*	*n.a.*	*n.a.*
CHE_DOF	0.371(0.039)	0.360(0.061)	0.229(0.051)	*n.a.*	*n.a.*	*n.a.*	*n.a.*	*n.a.*	*n.a.*	*n.a*
GBR_DOF	0.231(0.055)	0.590(0.120)	0.716(0.067)	*n.a.*	*n.a.*	*n.a.*	*n.a.*	*n.a.*	*n.a.*	*n.a.*
NOR_DOF	0.151(0.036)	0.356(0.069)	0.699(0.070)	*n.a.*	*n.a.*	*n.a.*	*n.a.*	*n.a.*	*n.a.*	*n.a.*
SRB_DOF	0.222(0.053)	0.514(0.105)	0.695(0.071)	*n.a.*	*n.a.*	*n.a.*	*n.a.*	*n.a.*	*n.a.*	*n.a.*
CZE_DOF & CHE_DOF	*n.a*	*n.a*	*n.a*	0.441(0.026)	0.186(0.029)	0.296(0.036)	0.378(0.026)	0.243(0.105)	0.088(0.030) 0.092(0.036)	0.390(0.045) 0.394(0.045)

*R^2^* of models was calculated as the ratio of the sum of the main and the interaction variance relative to the sum of the residual, the main and the interaction variance. n.a., not applicable.

### 3.4 Predictive ability for DMY

**Figure 1:**
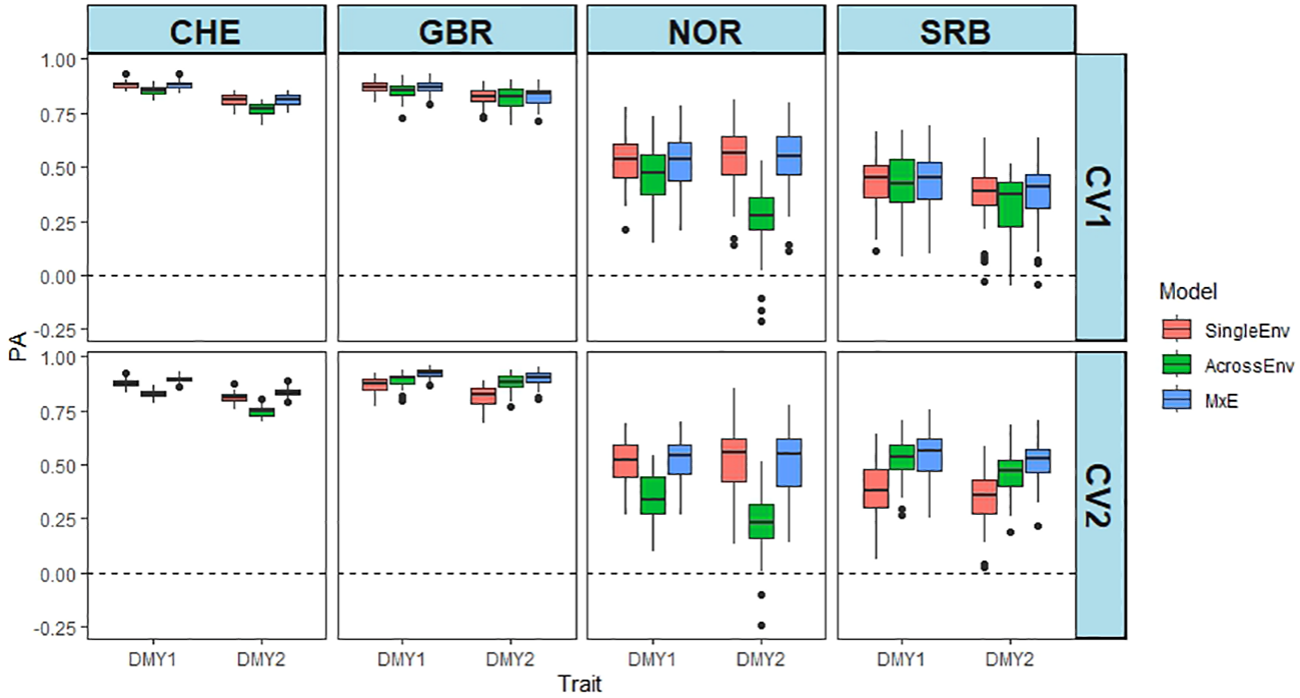
Predictive ability (PA) of DMY in year 1 and 2 (e.g. CHE_DMY1, CHE_DMY2 etc.) at four locations. PA is the Pearson correlation between predicted and the scaled and normalised BLUE values of DMY using SingleEnv, AcrossEnv and MxE models and CV1 and CV2 cross validation methods. The environmental comparisons are between year 1 and year 2 at each location separately. The boxplots represent 50 training-test partitions.

PA of the joint analysis of the annual DMY for two years at each location is shown in **Figure 1**. Analysis of variance of the DMY data showed that location_year, model and CV method, and their interactions had a significant effect on PA (**Supplementary Table S5**). The CV2 cross validation method resulted in higher PAs than CV1, and PAs from CHE and GBR were consistently high, while NOR and SRB gave lower and more variable PAs. The PA values varied between 0.232 in NOR_DMY2 AcrossEnv model and CV2 to 0.923 for GBR.1, MxE model and CV2 (**Supplementary Table S5**). Overall, the MxE model gave the highest PAs and AcrossEnv the lowest. The CV2 cross validation method generally resulted in higher PAs than CV1 (0.653 vs 0.638).

**Figure 2:**
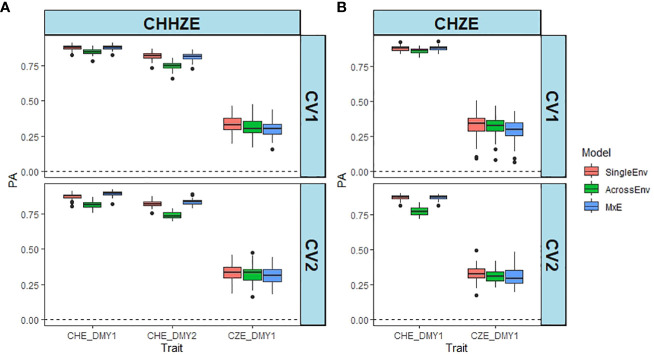
Comparison between predictive ability (PA) of DMY estimated in CHE and CZE. PA is the Pearson correlation between predicted and the scaled and normalised BLUE of DMY using SingleEnv, AcrossEnv and MxE models and CV1 and CV2 cross validation methods. **(A)** Comparison across three environments (CHHZE): CHE_DMY1, CHE_DMY2 and CZE_DMY1. **(B)** Pairwise comparison (CHZE) between CHE_DMY1 and CZE_DMY1. The boxplots represent 50 training-test partitions.

Both the three-way joint analysis (**Figure 2A**) and the pairwise joint analysis of CHE_DMY1 and CZE_DMY1 (**Figure 2B**) show that the PAs for CZE_DMY1 are much lower than those for CHE, irrespective of year, model and cross validation method (**Supplementary Tables S6**, **S7**). The MxE model resulted in the highest PA values, and the AcrossEnv model the lowest PA for CHE, while the SingleEnv gave the highest and the MxE interaction model, the lowest PA for CZE (**Supplementary Table S6**). The pairwise comparison between CHE_DMY1 and CZE_DMY1 in year 1 was similar, with the PAs for CHE varying between 0.774 and 0.879, while those for CZE_DMY1 were no higher than 0.331 (**Figure 2B**; **Supplementary Table S7**). Overall, the SingleEnv model resulted in the highest PA values and the AcrossEnv model gave the lowest PAs. There was no significant effect of the CV method used.

### 3.5 Predictive ability for CP content

**Figure 3:**
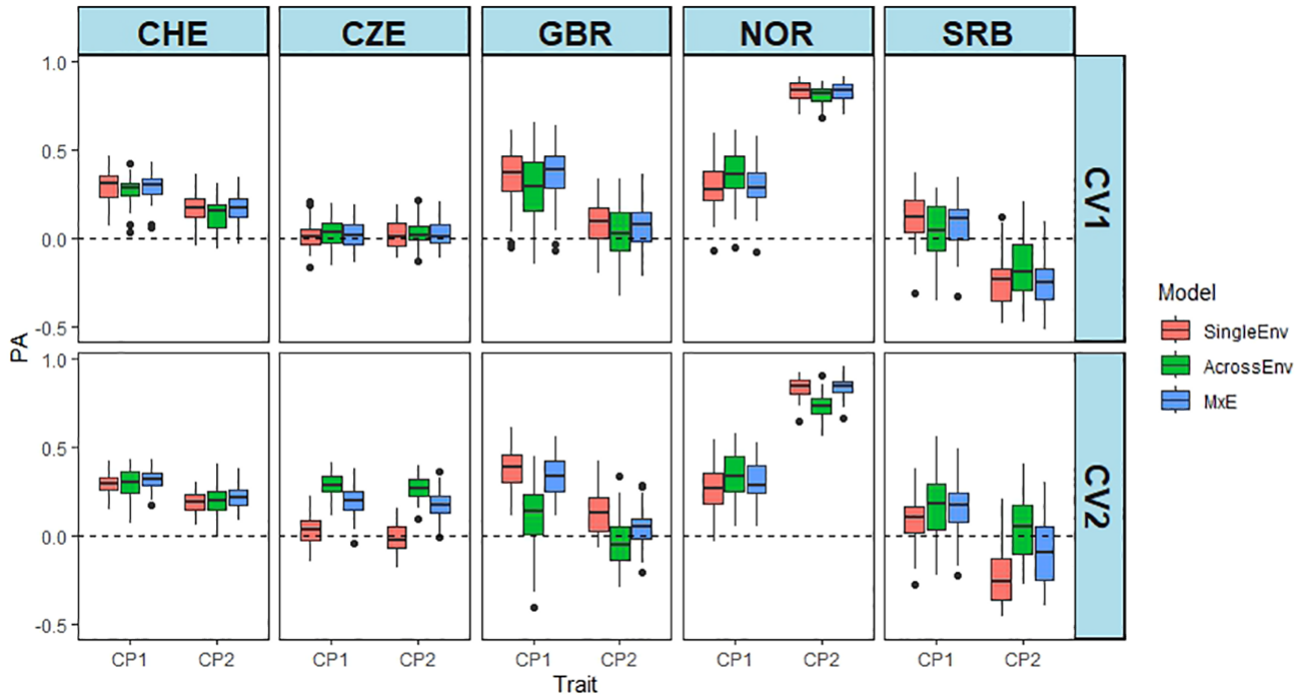
Predictive abilities (PA) of CP content from the joint analyses of cut 1 and cut 2 of year 1 at each location separately. PA is the Pearson correlation between predicted and the scaled and normalised BLUE values of CP content using SingleEnv, AcrossEnv and MxE models and CV1 and CV2 cross validation methods.

The PAs for the CP content were generally much lower than for DMY, except for NOR_CP2 (**Figure 3**; **Supplementary Table S2**), for which a higher proportion of the variance was explained by the marker main effect (**Table 3**; **Supplementary Figure S4**). PA from NOR_CP2 varied between 0.733 and 0.836, but were low, even zero or negative for the other Location_cut combinations. Overall, the MxE model performed best, while the SingleEnv model gave the lowest PAs (**Supplementary Table S8**). The CV2 method was slightly superior to CV1. The multi-environment analysis of all four environments in CHE and CZE showed that CHE_CP1 had the highest PA (**Supplementary Figure S5**), while CZE_CP1 and CZE_CP2 both resulted in PA values around zero. The MxE model performed best, and the AcrossEnv model was poorest. Overall, the CV2 method resulted in higher PA values than CV1 (**Supplementary Table S9**). The PAs from CHE_CP1 had low to moderate PAs, while the PAs for CZE were around zero for both CV methods (**Supplementary Table S9**). The SingleEnv and the MxE interaction models were better than AcrossEnv. The mean PA of the CV2 method was slightly higher than CV1.

### 3.6 Predictive ability for DOF

**Figure 4:**
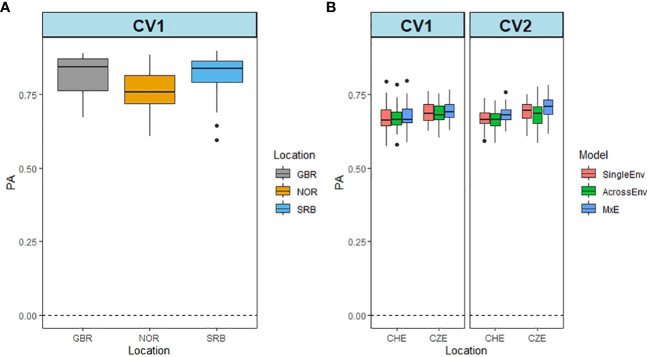
Predictive ability of flowering time (DOF) from Britain (GBR), Norway (NOR) and Serbia (SRB) using the SingleEnv analysis and the CV1 cross validation method **(A)**. Prediction ability from CHE and CZE using SingleEnv, AcrossEnv and MxE models and CV1 and CV2 cross validation methods **(B)**.

DOF was recorded in year 1 only, so the data from GBR, NOR and SRB were analysed by the SingleEnv model. **Figure 4A** shows that the PA values were high for all three locations, varying between 0.762 in NOR and 0.838 in GBR (**Supplementary Table S10**). For CHE and CZE, a pairwise comparison between the two locations was carried out, using all three models and both cross validation methods. In both locations the PA was high (0.671 for CHE, and 0.690 for CZE) (**Figure 4B**; **Supplementary Table S11**). The MxE model again performed better than SingleEnv and AcrossEnv. There were no significant differences between CV methods. The high PA values are in line with the high heritability values recorded for DOF (**Supplementary Table 2**) (Nay et al., 2023).

### 3.7 Prediction bias

**Figure 5:**
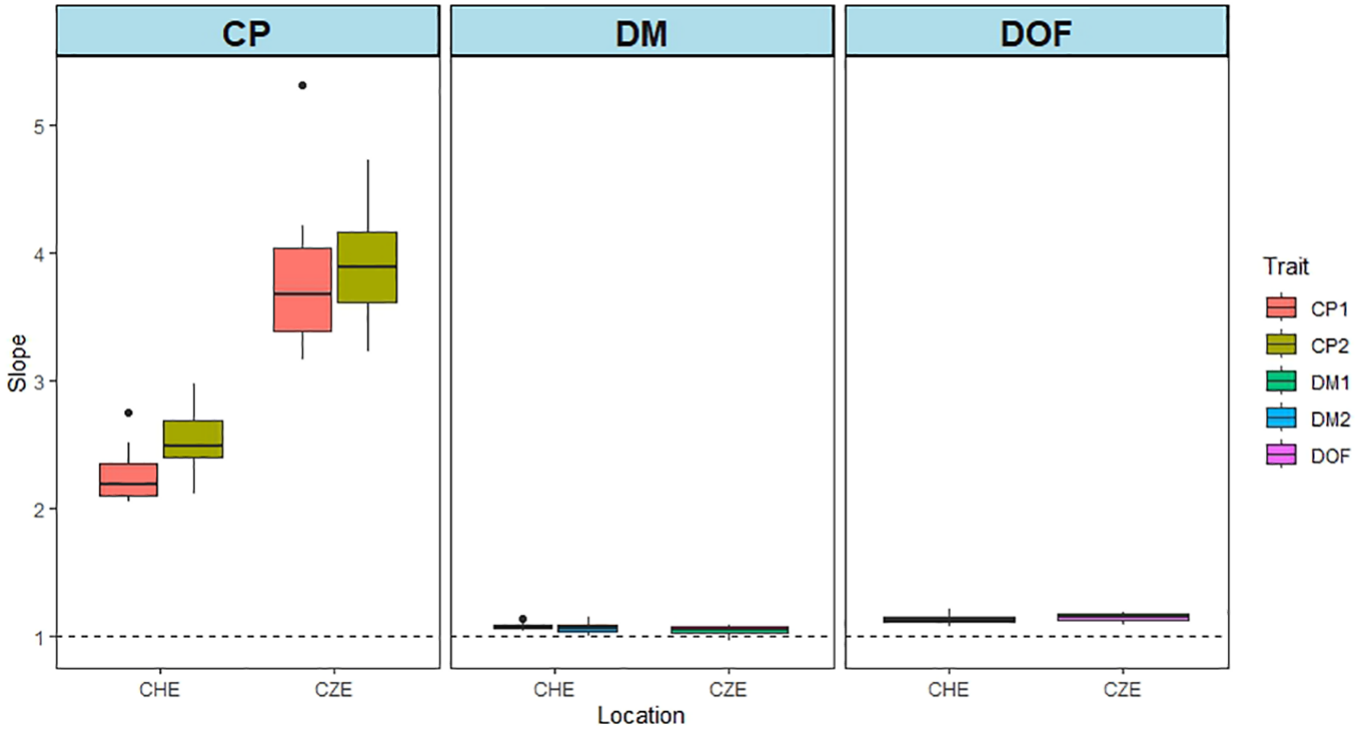
Bias of predictions for data from CHE and CZE. The graphs show the slopes of the regression of GEBVs on phenotypic values. The stippled line indicates a slope value of 1. The GEBVs are based on the joint analysis of all the environments for each of the three traits: CP content, DMY and DOF. The MxE interaction model and the CV2 cross validation method were used. Fifty iterations of training-test set combinations were performed. CP1 and CP2 represents CP content of cut 1 and cut 2 in year 1, respectively. DMY1 and DMY2 represent annual DMY in year 1 and year 2, respectively.

Possible biases of predictions can be revealed by regressing the observed phenotypes on the GEBV. The results of this analysis are shown in **Figure 5**. We used the MxE interaction model and the CV2 cross validation method on the data from CHE and CZE. A slope of 1 indicates no bias. The larger the deviation from 1, the larger the bias. For CP content the bias was largest (3.13), while for DMY (1.07) and DOF (1.15), the slope values were near 1, indicating low bias (**Supplementary Table S12**). This is consistent with PA values, which were generally high for DMY and flowering, and low for CP overall (**Figures 1**–**4**).

### 3.8 Effect of marker numbers and training set size on predictive ability

**Figure 6:**
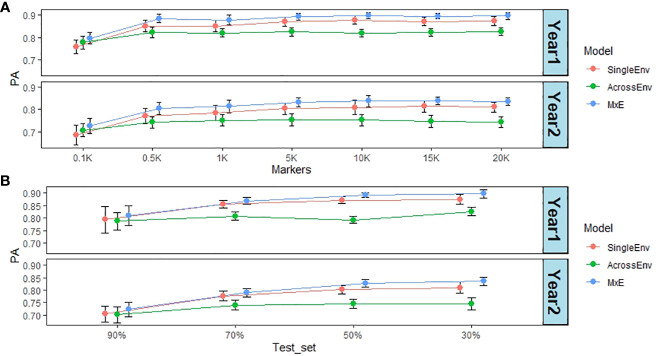
**(A)** Effect of marker numbers (in thousands) on prediction ability (PA). The default training set/test set ratio of 70%/30% was used; **(B)** Effect of test set percentage on PA. The full set of 20,156 markers were used. For both analyses DMY from year 1 and 2 in CHE were used as phenotypes, together with the CV2 cross validation method.

The effect of marker numbers on the PA values was investigated by sampling subsets of the full marker set of 20,156 SNPs (20K), down to 100 SNP markers (0.1K). The PA values are based on DMY data from year 1 and 2 in Switzerland (CHE_DMY1 and CHE_DMY2). The CV2 method of cross validation was used, as it was performing best throughout this work. Remarkably, the effect of lowering the marker numbers was not dramatic until less than 1000 markers were used (**Figure 6A**; **Supplementary Table S13**). Overall, PA was reduced from 0.83 at 10K, 15K and 20K markers to 0.74 at 100 markers (0.1K), still high for such a small number of markers. At all marker numbers, the MxE model performed best (0.85), and AcrossEnv performed worst (0.78) (**Supplementary Table S13**).

The effect of the percentages of all accessions used as the test set was also analysed. The default split used throughout this work was 70% as training set and 30% as test set. **Figure 6B** summarises the results obtained by increasing the test set percentage from 30% to 90%, and therefore decreasing the training set percentage from 70% to 10%. There was a significant effect of test set percentage with the smallest (30%) having the highest PA of 0.83 and the largest (90%) having the lowest PA of 0.75 (**Supplementary Table S14**). Again, the MxE interaction model gave the highest PA values, and AcrossEnv the lowest. Visual inspection of the data (**Figure 6B**) suggests that the decrease in PA takes effect when the test set percentage is larger than 50%.

**Figure 7:**
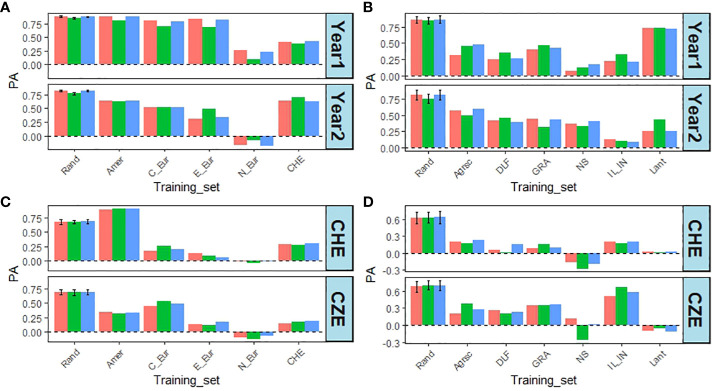
Effect of leaving one region or one breeders’ material out from the training set for DMY, from CHE in year 1 and 2, and DOF from CHE and CZE in year 1. **(A)** PA for DMY when leaving one region out. **(B)** PA for DMY when leaving out one breeders’ material. **(C)** PA for DOF from CHE or CZE, when leaving one region out. **(D)** PA for DOF from CHE or CZE when leaving out one breeders’ material. Rand – Mean and standard deviation of 10 random removals of 142 accessions (regions) or 61 accessions (breeders’ material) using the CV1 method; Amer – Americas; C_eur – Central Europe; E_eur – Eastern Europe; N_eur – Northern Europe; CHE – Switzerland; Agrsc – Agroscope; DLF – DLF; GRA – Graminor; NS – IFVCNS; IL_IN – ILVO_INRAe; Lant – Lantmännen. Bar colours: red = SingleEnv; green = AcrossEnv; blue = MxE.

The effect of removing accessions from specific regions or specific breeders’ material was more pronounced for the latter (**Figure 7**). There was also a larger reduction in PAs for DOF than for DMY. Concerning the regions removing Northern European accessions resulted in the largest reduction in PA compared to the random control for both traits, but removing Swiss accessions also had a sizeable effect (**Figures 7A, C**). For DMY, removing the Lantmännen breeders’ material had the smallest effect on PA, at least in year 1, while it reduced PA to zero or negative values for DOF (**Figures 7B, D**).

### 3.9 Test of predictive ability on new phenotypic data

**Figure 8:**
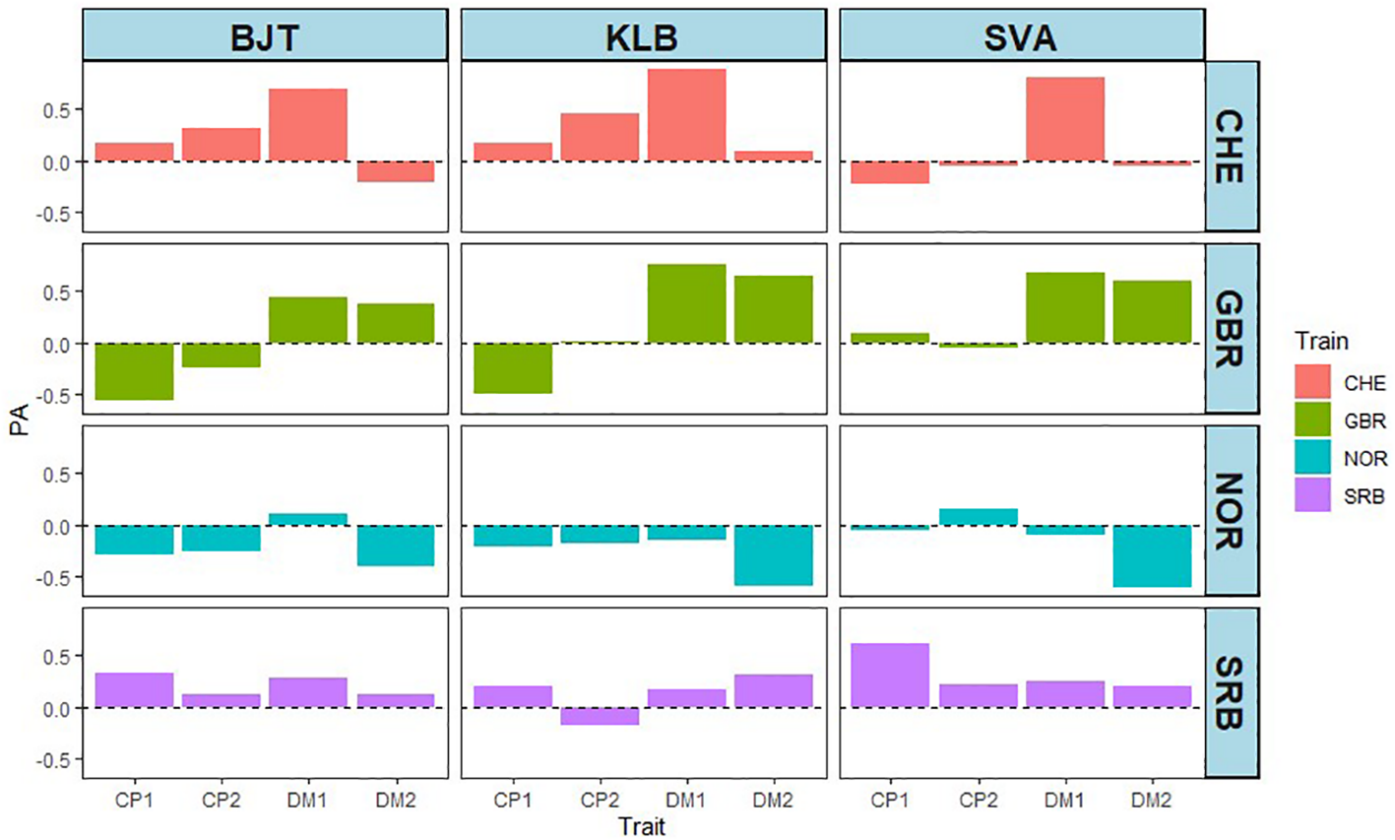
Effect of training sets from CHE (392), GBR (99), NOR (107) and SRB (100) on prediction ability (PA) at three locations in Sweden (BJT = Bjertorp; KLB = Kölbäck; SVA = Svalöv) for DMY year 1 and 2, (DM1 and DM2) and CP in cut 1 and cut 2 in year 1 (CP.1 and CP.2).

So far, the prediction abilities presented here are based on cross validations derived from training and test sets where both phenotypic and genotypic data from the same accessions are used by the models. To test the predictions on new phenotypic data not included in any of the analyses, we used DMY and CP content from field trials carried out at three locations in Sweden (BJT, KLB, SVA; **Supplementary Figure S1**). A total of 42 accessions from the EUCLEG panel were used. The DMY and CP content data are shown in **Supplementary Table S3** and in **Supplementary Figures S6** and **S7**. The broad sense heritability for each of the traits revealed that DMY traits had high heritability, while CP content had low to medium heritability (**Supplementary Table S4**). This is broadly in agreement with the results from the other five locations. The PAs for the four traits at the three locations are shown in **Figure 8**. There are four sets of PAs per location in Sweden, and they represent the origin of the training set. Each PA value is the correlation between the phenotypic value of the trait in one of the Swedish locations and the GEBV of the corresponding trait from one of the four other countries. Using NOR data as training sets gave rise to uniformly low PAs, which were either negative or close to zero. Data with GBR training sets gave low or negative values for CP content, but moderate to high PA values for DMY, particularly in year 1. The SRB training sets gave rise to low or poor values throughout with only CP1 at SVA being high. The CHE training set was the largest (392 accessions) and gave low to moderate PA values for CP content, except for SVA, and high prediction ability for DMY in year 1, but not year 2. These results confirm our earlier results with DMY giving higher PA than CP content, and they are overall in line with the contrasting heritability for the two traits.

## 4 Discussion

In our work on red clover presented here the MxE model outperformed the SingleEnv and AcrossEnv models. The MxE model was initially developed and tested using wheat yield data by Lopez-Cruz et al. (2015). They showed that phenotypic correlation between pairs of environments was directly associated with the proportion of genomic variance explained by the main effect of the markers. They also demonstrated that the MxE model fitted the data better than the AcrossEnv model. In the vast majority of cases prediction accuracy was highest when the MxE model was used.

Similar results were obtained in an analysis of agronomic traits in sesame (Sabag et al., 2023). However, only two environments were compared (two consecutive years), and the phenotypic correlation for all nine traits tested were all rather high between years (0.50 to 0.96, depending on the trait). While the trait with the lowest correlation also had the lowest PA, it also had the lowest heritability, so it is difficult to disentangle the effects of the two factors. Nevertheless, it seems that the results described here for red clover follow the same trends.

### 4.1 Variance components

The proportion of total variance explained by the main marker effects was a good indicator of correlation between environments. This was the case for DMY and DOF, where the pairwise correlation between phenotypic values associated with the main effect variance proportion (**Supplementary Figure S3**; **Supplementary Table S2**). This is similar to what was found by others (Lopez-Cruz et al., 2015; Crossa et al., 2016; Bandeira E Sousa et al., 2017; Sabag et al., 2023). For DMY in year 1 in CZE however, the variance due to the main marker effect was small, while the environment-specific effect from the MxE model was very large (**Table 1**; **Supplementary Figure S3**). The model thus explains a large proportion of the phenotypic variance, but most of it is environment specific. It may be in this environment that many markers with environment-specific effects will be found. Crossa et al. (2016) also observed that in complex traits with lower heritability, such as grain yield and grain density, a larger fraction of the total genetic variance was due to environment-specific effects. This contrasted with the heading date and thousand grain weight, traits for which heritability was high and the environment-specific proportion of the genetic variance was low. This is consistent with the low heritability observed for DMY in year 1 in CZE (**Supplementary Table S2**).

The variance component analyses showed that the MxE model for the red clover data gave a better fit to the data than the AcrossEnv model (**Tables 1**–**3**), similar to what was found in wheat (Lopez-Cruz et al., 2015; Crossa et al., 2016) and maize (Bandeira E Sousa et al., 2017). In sesame, the AcrossEnv model was not included in the analyses, but the MxE model was found to enhance the PAs relative to the SingleEnv model (Sabag et al., 2023).

### 4.2 Predictive ability

Overall, the PAs were high for DMY and DOF, and low for CP content. This follows the heritability for the traits (**Figures 1**–**4**; **Supplementary Table S2**). The high phenotypic correlation between year 1 and year 2 of DMY in GBR (0.87) and CHE (0.71), respectively, may explain why the PA for those traits were uniformly high in both years (0.83 – 0.91) for the MxE model and CV2 cross validation method.

For CP content an exception was observed for the PA for NOR in cut 2, which was 0.83 for the SingleEnv and MxE models (**Supplementary Table S8**), and the heritability for this specific CP trait was also high (**Supplementary Table S2**). It is unclear whether this aberrant result is connected to the notion that most of Northern European accessions tend to have been adapted to one cut per season. The phenotypic correlation between NOR_CP1 and NOR_CP2 was 0.35, and this is probably why the PAs for cut 1 (0.26 – 0.37) were significantly lower than for cut 2 (**Supplementary Table** **S2**).

The MxE model performed better than the AcrossEnv model when pairs of environments were analysed jointly. This was the case for both DMY and CP content (**Supplementary Tables S5**, **S8**). The superiority of the MxE model was more pronounced when the CV2 cross validation method was used, and for pairs of environments with high phenotypic correlation (**Figures 1**–**3**; **Supplementary Table S2**). This is because the genetic covariance between environments is forced to be positive, as it is a product of the variance of the main effects (Lopez-Cruz et al., 2015). This also makes sense, because the CV2 method allows for borrowing information about accessions tested in one environment, but not in another. If the correlation is high, the information borrowed will be more accurate than if the correlation is low (Lopez-Cruz et al., 2015; Crossa et al., 2016; Sabag et al., 2023). In contrast, when CV1 is used for cross validation, the same accessions are missing from environments being analysed jointly. The SingleEnv analysis would then be expected to perform similarly to the AcrossEnv and MxE models. This was the case in red clover, at least for DMY (**Figure 1**), when correlations between pairs of traits were moderate to high. It should also be noted that for the SingleEnv analyses, the distinction between CV1 and CV2 is meaningless.

Overall, PAs for DMY were high (**Figures 1**, **4**; **Supplementary Tables S5**-**S7**). Particularly, CHE and GBR had high PA for DMY (0.80–0,88) and for DOF (0.67 – 0.84) (**Figure 4**; **Supplementary Tables S10**-**S11**). To the best of our knowledge, no GP work has been published yet for red clover to compare directly with our study. A PA of 0.30 for biomass yield was reported for white clover (Griffiths et al., 2022). Despite such a comparatively low accuracy, selection based on the GEBVs resulted in higher gain than phenotypic selection. In alfalfa, Annicchiarico et al. (2015) reported PA values of 0.32 – 0.35 for biomass yield in two populations, while Jia et al. (2018) found PAs of 0.13 for biomass yield in a diverse alfalfa germplasm collection. In breeding populations, PAs were found to vary between 0.21 – 0.66 depending on breeding cycle and location (Li et al., 2015). Recently, Pégard et al. (2023) reported a mean PA of 0.65 for DMY from two locations, France and Serbia, and 0.41 for DOF in a diverse panel of alfalfa accessions, where the marker data also were obtained as allele frequencies derived from sequencing of pooled plant material representing each accession. The PA values for DMY reported in the present work thus compare well with those reported for alfalfa and white clover.

Prediction abilities for DMY in the perennial ryegrass forage crop were 0.013 – 0.275 (Grinberg et al., 2016), 0.07 – 0.43 (Faville et al., 2018), and 0.28 – 0.59 (Pembleton et al., 2018). Despite low PAs on DMY, Faville et al. (2020) found that genomic prediction could still be used to improve the trait. It should be noted that these values were obtained using breeding populations, i.e. likely narrower germplasm, than in the present work. The germplasm used here was a diverse panel with significant population structure (Nay et al., 2023), which can inflate PA (Guo et al., 2014; Werner et al., 2020). **Figure 7** demonstrates how this affected PAs of DMY and DOF. By having all accessions representing e.g. a single breeder in the test set and not in the training set, the PA values were mostly affected negatively. This is likely because no accessions from such a relatively narrow set were represented in the training population, whereas for the random CV, those accessions were present in both sets (Werner et al., 2020). There were variations in the magnitude of the effect depending on the trait, year (DMY) or location (DOF). Some of this may be explained by differences in the genetic architecture of the traits. The Northern European accessions tend to have been adapted to one cut per season, and late flowering, and may thus be genetically distinct from the other accessions, which are early flowering and adapted to a multi-cut management regime, hence the low PA for DMY (**Figure 7A, C**). There was a more modest decline for the two Northern European breeding materials, Graminor, and Lantmännen. This could possibly be explained by the presence of one of the two in either the training set and the test set (**Figures 7B, D**).

The PA values were remarkably resilient to lowering the number of SNP markers used to obtain the genomic relationship matrix (GRM) (**Figure 6**; **Supplementary Table S13**). While the PA values for marker numbers at 1K and below were significantly reduced, the PA value for 100 markers were still 0.74. The most likely explanation is that the genomic relationship does not need many markers to be reasonably stable. The GRM was obtained according to VanRaden (2008) for the GBLUP model. It has been reported that a GRM matrix, derived from the Euclidian distance between individuals based on the markers, can better capture non-additive marker interactions (Cuevas et al., 2016; Bandeira E Sousa et al., 2017). This remains to be tested in red clover. There are also other ways of modelling the GxE effect to better predict the performance of accessions across environments. Incorporating a factor analytic (FA) structure accommodates different trial designs and unbalanced data, allows for heterogeneity of variance across environments, and can help to explain GxE interaction in terms of a few latent factors (common factors) affecting the performance of genotypes across environments (Smith et al., 2001; Smith and Cullis 2018). This should be explored in future work.

### 4.3 Validating the training sets

**Figure 9:**
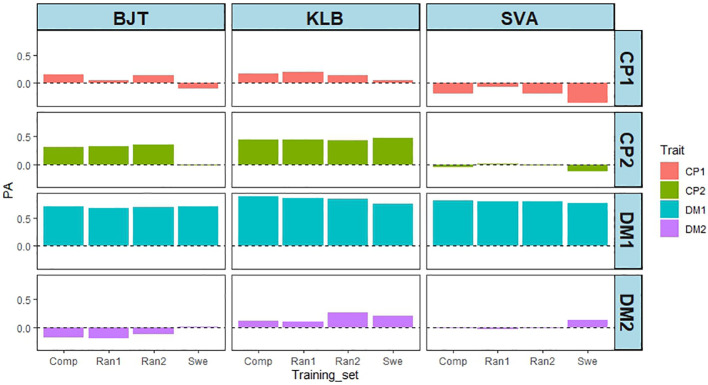
Effect of varying the training sets from CHE on PA from three locations in Sweden (BJT = Bjertorp; KLB = Kölbäck; SVA = Svalöv) for DMY in year 1 and 2, (DMY1 and DMY2) and CP content in cut 1 and cut 2 in year 1 (CP1 and CP2). Training sets were: Comp – Complete set of 392 accessions; Swe – Training set without the 42 accessions tested in Sweden; Ran1 and Ran2 – Training sets with 42 accessions removed randomly.

Given the geographical proximity of field trial sites, it was surprising to find that the NOR training set predicted the phenotypes obtained in Swedish field trials so poorly. The CHE training set was the largest (392 accessions), and it contained 41 of the 42 EUCLEG accessions tested in Sweden. To ascertain whether this overlap was important, three training sets were compared with the complete (Comp, 392 accessions) set: a training set in which the 42 accessions tested in all three locations in Sweden were omitted, and two training sets in which 42 accessions were removed randomly (Ran1 and Ran2). **Figure 9** shows that reducing the size of the training set by 42 did not appear by itself to have any effect on PA, but removing the 42 accessions tested in Sweden did lower the PA for CP1, and particularly for CP2 in BJT (0.32 - -0.01). For DMY in year 1 there was also a slight decrease of PA in KLB from 0.84 to 0.75, and in SVA from 0.80 to 0.77. A similar analysis was not feasible for the GBR, NOR and SRB training sets, as they were much smaller and had varying numbers of accessions overlapping with the 42 from Sweden (3 from GBR, 5 from SRB, and 22 from NOR).

**Figure 10:**
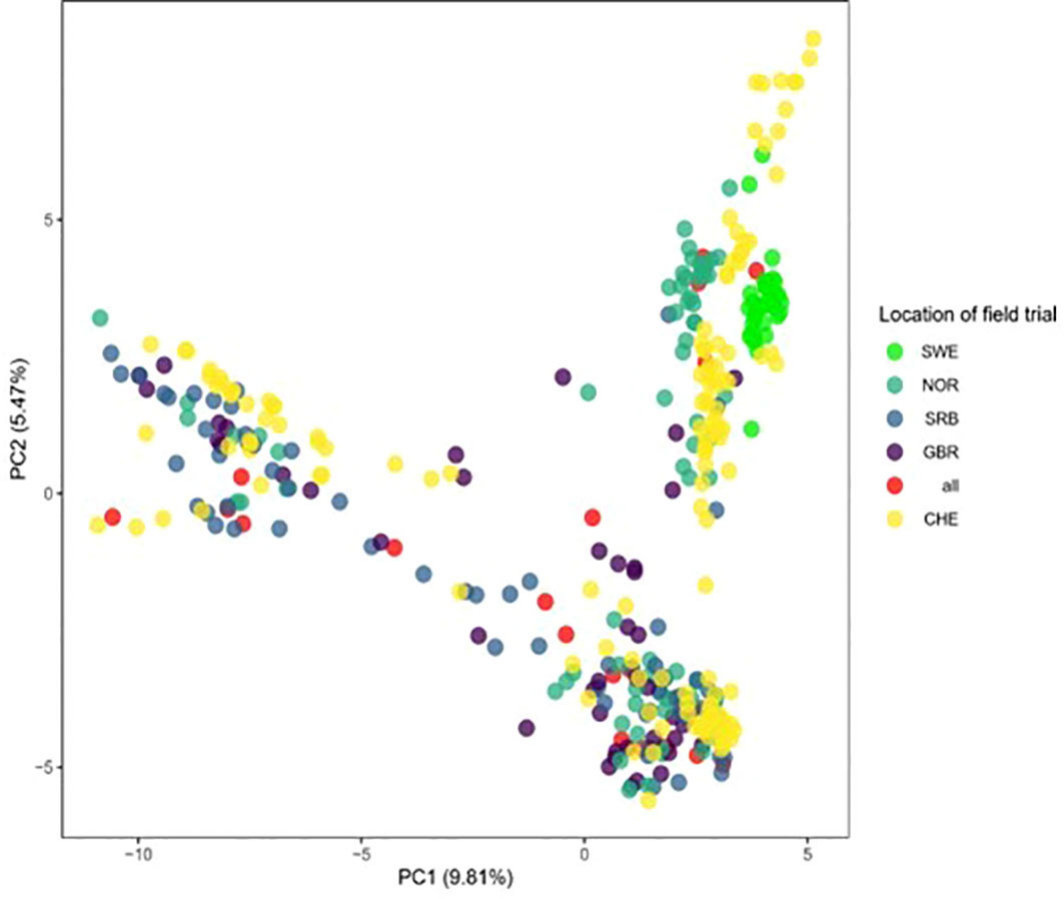
Principal component analysis (PCA) revealing the genetic relationship between the 392 accessions used in this work. CHE: Accessions that were exclusively assessed in Switzerland; GBR: Accessions exclusively assessed in GBR and CHE; NOR: Accessions exclusively tested in NOR and CHE; SRB: Accessions exclusively tested in SRB and CHE; all: Accessions tested in CHE, GBR, NOR and SRB; SWE: Accessions tested exclusively in Sweden and CHE.

The genetic relationship between training set and test set is important for accuracy of genomic predictions (Liu et al., 2015). We used the principal component analysis (PCA) carried out by Nay et al. (2023) to illustrate the genetic relationship between the training sets with accessions from CHE, GBR, NOR and SRB with the test set from Sweden (**Figure 10**). There is a sizeable number of accessions tested in NOR that are closely related with the accessions tested in Sweden (in addition to the 22 accessions that are in common with those tested in Sweden). In contrast, the GBR and SRB accessions are more dispersed among the 392 accessions. Together with the results shown in **Figure 9**, it suggests that genetic relationship *per se* does not explain why the NOR training set predicted the phenotypes obtained in the Swedish field trials so poorly. What might explain it is the effect of leaving one breeders’ material out. Leaving out the Lantmännen breeders’ material does not reduce the PA by much relative to the random control (0.85 to 0.77) in year 1 (**Figure 7B**), which could be explained by the presence of related accessions in both training and test set. However, that does not explain why the PAs from year 2 reduced to 0.31 on average. Factors related to the environment and the genetics of the trait must be playing a role.

Overall, the present work has demonstrated the importance of correlation between phenotypic traits for PA. We therefore investigated the correlation between the phenotypic data from the populations from CHE, GBR, NOR and SRB with the test sets from Sweden. This could only be ascertained fully using the CHE population as it encompassed 41 of the 42 accessions tested in Sweden. **Table 4** shows that DMY in year 1 was the only trait for which there was a significant positive correlation between the phenotypic values in Sweden and the corresponding phenotypic values from CHE. This was also the only trait for which the PA was very high. This would appear to underline the importance of phenotypic correlation for PA.

**Table 4:** Pearson correlation between phenotypes from the three Swedish locations (BJT, Bjertorp; KLB, Kölbäck; SVA, Svalöv) and the phenotypic data from the corresponding CHE traits.

	BJT		KLB		SVA	
Trait	Year 1	Year 2	Year 1	Year 2	Year 1	Year 2
DMY1	0.577***		0.824***		0.698***	
DMY2		-0.447		-0.652		-0.749
	Cut 1	Cut 2	Cut 1	Cut 2	Cut 1	Cut 2
CP1	0.131		0.129		-0.035	
CP2		-0.333		-0.243		0.052

Correlations with statistically significant (*P< 0.001*) positive values are indicated with ***.

## 5 Conclusions

This work is the first evaluation of GP in red clover. It shows that PAs were high for DMY and DOF, but mostly low for CP content. The results probably reflect differences in heritability, prediction bias, and correlation between traits. A lower number of markers in the models resulted in lower PAs, but only when they dropped below 1000 markers. Similarly, increasing the test set size at the expense of the training set size also reduced PA, but only when the training set size dropped to 10%. Such high PA values may be caused by the population structure present in the diverse red clover panel used here, because genetically related accessions are present in both training and test sets. Another important factor enhancing PAs seems to be a positive correlation between phenotypic traits in the training and test sets.

The prediction models incorporated GxE by capturing MxE interaction effects, which overall enhanced PA. This has perspectives for identifying markers with effects that are stable across environments, and those that have environment-specific effects.

## Data availability statement

The datasets presented in this study can be found in online repositories. The names of the repository/repositories and accession number(s) can be found below: https://www.ncbi.nlm.nih.gov/, PRJNA842231.

## Author contributions

LS: Conceptualization, Formal Analysis, Investigation, Methodology, Validation, Writing – original draft, Writing – review & editing, Funding acquisition. MMN: Formal Analysis, Investigation, Writing – review & editing. CG: Conceptualization, Formal Analysis, Investigation, Methodology, Writing – review & editing. LAF: Formal Analysis, Investigation, Writing – review & editing, Conceptualization, Data curation, Validation. MP: Formal Analysis, Investigation, Methodology, Writing – review & editing. LO: Data curation, Investigation, Methodology, Writing – review & editing. HA: Investigation, Writing – review & editing, Data curation, Resources. JR: Investigation, Writing – review & editing. LJ: Investigation, Writing – review & editing. AP: Conceptualization, Data curation, Resources, Writing – review & editing. TR: Writing – review & editing, Formal Analysis, Investigation, Methodology. DL: Funding acquisition, Investigation, Writing – review & editing. CH: Funding acquisition, Resources, Supervision, Writing – review & editing. RK: Data curation, Formal Analysis, Funding acquisition, Investigation, Validation, Writing – original draft, Writing – review & editing.
