# Supplementary material for: Including marker x environment interactions improves genomic prediction in red clover (Trifolium pratense L.)
Source: Front Plant Sci. 2024 Jun 10;15:1407609. doi: 10.3389/fpls.2024.1407609 (PMC11194335; doi:10.3389/fpls.2024.1407609)
Supplement: Supplementary file 1 [file DataSheet_1.zip › Supplementary_Material_Red_Clover_GS_manuscript_revision2.docx]

Supplementary Material

**Including marker x environment interactions improves genomic prediction in red clover (*Trifolium pratense* L.)**

Leif Skøt^1^*, Michelle Nay^2^, Christoph Grieder^2^, Lea Frey^3^, Marie Pégard^4^, Linda Öhlund^5^, Helga Amdahl^6^, Jasmina Radovic^7^, Libor Jaluvka^8^, Anna Palmé^9^, Tom Ruttink^10,11^, David Lloyd^12^, Catherine J. Howarth^1^, Roland Kölliker^3^*

*** Correspondence:** Corresponding Authors: [lfs@aber.ac.uk](mailto:lfs@aber.ac.uk); [roland.koelliker@usys.ethz.ch](mailto:roland.koelliker@usys.ethz.ch)

# Supplementary Figures and Tables

## Supplementary Tables

Supplementary_Table1.xlsx

Supplementary_Table2.xlsx

Supplementary_Table3-4.xlsx

Supplementary_Table5-14.xlsx

**1.2 Supplementary Figures**


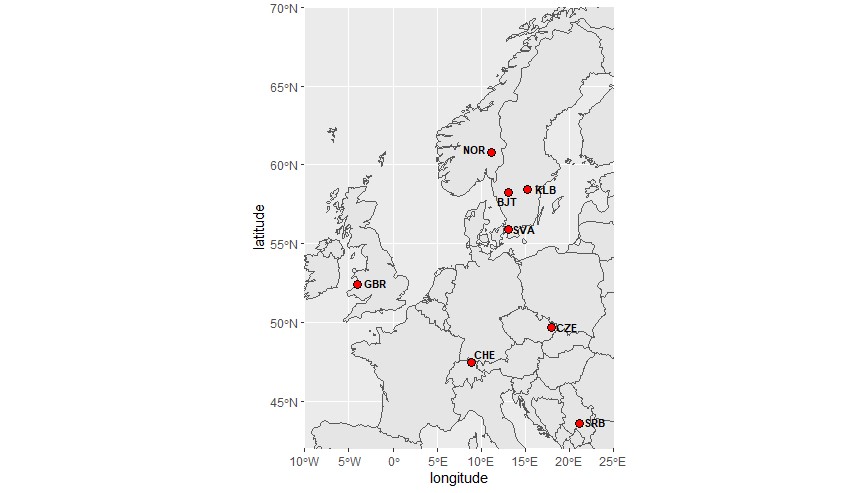


**Supplementary Figure 1.** Location of the sites where the red clover field trials took place. CHE: Agroscope, Switzerland; CZE: DLF, Czechia; GBR: IBERS, Britain; NOR: Graminor, Norway; SRB: Institute for Forage Crops, Serbia; BJT: Bjertorp, Sweden; KLB: Kölbӓck, Sweden; SVA: Svalöv, Sweden

**CV1**

**CV2**

Environment 1

Environment 2

Environment 1

Environment 2

**Supplementary Figure 2**. Cross validations: Blue = training population, Green = test population. CV1: Test accessions have not been assessed in any of the environments. CV2: Test accessions have been evaluated in some, but not all environments. The default training/test population ratio was 70/30.


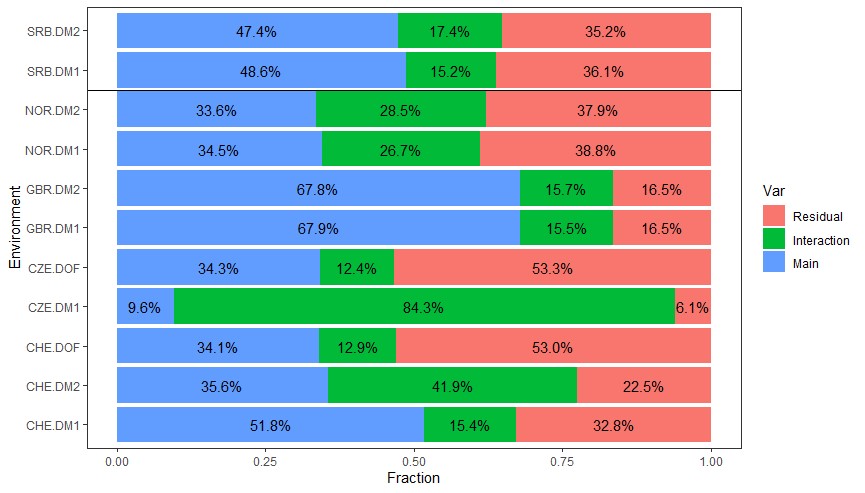


**Supplementary Figure 3.** Proportion of the total variance for DM yield and DOF attributed to the main marker variance (common between environments), environment-specific marker effect, and residual variance in the MxE model.


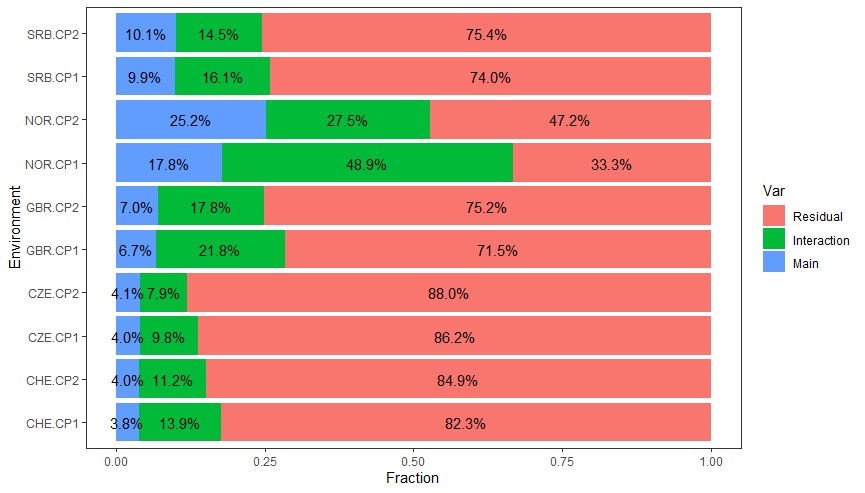


**Supplementary Figure 4.** Proportion of the total variance for CP content attributed to the main marker variance (common between environments), environment-specific marker effect, and residual variance in the MxE model.


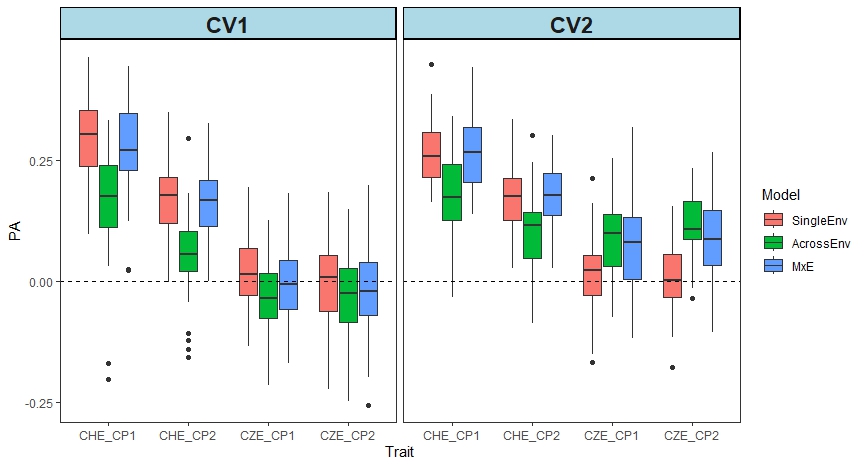


**Supplementary Figure 5.** Predictive ability (PA) of CP content data based on the joint analysis of all four environments in CHE and CZE. PA is the Pearson product moment correlation between predicted and the scaled and normalised BLUE values of CP content using the SingleEnv, the AcrossEnv or the MxE model with two cross validation methods (CV1 and CV2).


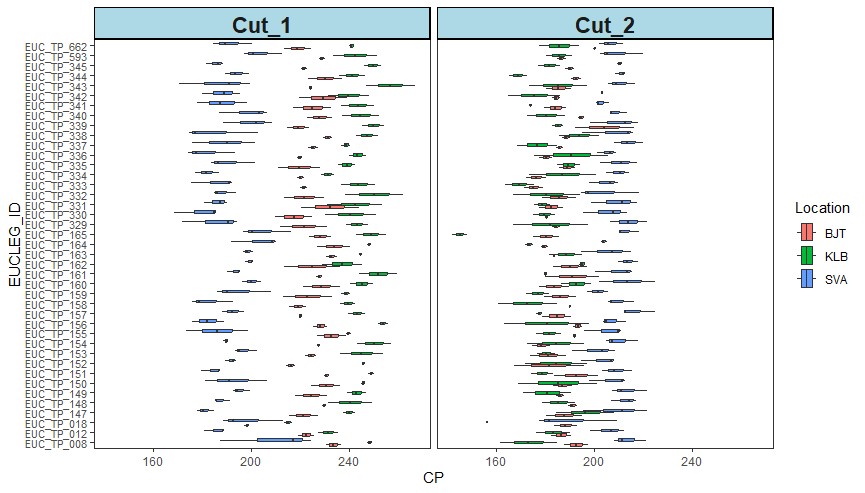


**Supplementary Figure 6.** Summary of CP content from three locations in Sweden: BJT = Bjertorp, KLB = Kölbӓck, SVA = Svalöv. CP is mg kgDM^-1^.


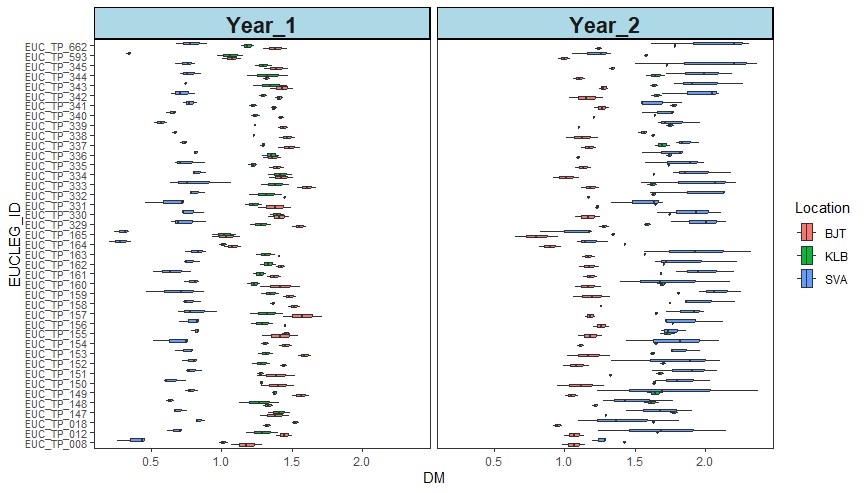


**Supplementary Figure 7.** Dry matter (DM) yield data from three locations in Sweden**:** BJT = Bjertorp, KLB = Kölbӓck, SVA = Svalöv. The DM yield is kg m^-2^ y^-1^. Y1 is year 1, and Y2 is year 2.
